# Reply to Morone, G.; Giansanti, D. Comment on “Anwer et al. Rehabilitation of Upper Limb Motor Impairment in Stroke: A Narrative Review on the Prevalence, Risk Factors, and Economic Statistics of Stroke and State of the Art Therapies. *Healthcare* 2022, *10*, 190”

**DOI:** 10.3390/healthcare10050847

**Published:** 2022-05-05

**Authors:** Saba Anwer, Asim Waris, Syed Omer Gilani, Javaid Iqbal, Nusratnaaz Shaikh, Amit N. Pujari, Imran Khan Niazi

**Affiliations:** 1 School of Mechanical & Manufacturing Engineering, National University of Sciences and Technology (NUST), Islamabad 45200, Pakistan; sanwer.bmes19smme@student.nust.edu.pk (S.A.); asim.waris@smme.nust.edu.pk (A.W.); omer@smme.nust.edu.pk (S.O.G.); j.iqbal@ceme.nust.edu.pk (J.I.); 2 Faculty of Health & Environmental Sciences, Health & Rehabilitation Research Institute, AUT University, Auckland 0627, New Zealand; nusrat.shaikh@aut.ac.nz; 3 School of Physics, Engineering and Computer Science, University of Hertfordshire, Hatfield AL10 9AB, UK; amit.pujari@ieee.org; 4 School of Engineering, University of Aberdeen, Aberdeen AB24 3FX, UK; 5 Center of Chiropractic Research, New Zealand College of Chiropractic, Auckland 1060, New Zealand; 6 Center for Sensory-Motor Interaction, Department of Health Science & Technology, Aalborg University, 9000 Alborg, Denmark

Thank you so much for your kind remarks. We really appreciate your detailed analysis [1] of this review study [2]. The use of robotics based on the concept of artificial intelligence in the field of rehabilitation is really an interesting subject, and it will be a pleasure for us to give an opinion on this issue.

Firstly, it is very important to understand that, with the increasing rate of stroke-related disability, it will be difficult to provide stroke survivors with post-stroke care (PSC) services because of their unbearable economic consequences, which raises the need to minimize the role of physical therapists and move towards adopting self-rehabilitative home-based therapies to facilitate healthcare for those living in remote areas.

Secondly, the rehabilitation field is shifting from conventional approaches to new and technologically advanced therapeutic strategies in which virtual reality, telerehabilitation, robotics, and invasive and non-invasive stimulations are top of the list.

For the use of artificial intelligence to fabricate modern medical equipment such as prosthetic limbs, robotic machinery is inevitable. Such systems are strongly bound with the commitment to making rehabilitation systems more comfortable, with a significantly increased degree of freedom for stroke patients or victims of limb amputations.

As An example, I will mention a commercially available system of Saebo-VR (https://www.saebo.com/virtual-reality/) (accessed on 28 March 2022) which is an interactive, multisensory computer-based simulation environment providing the patients with an opportunity to perform different activities in the real world. Saebo also provides a cutting-edge Saeb Glove (https://uk.saebo.com/shop/saeboglove/) (accessed on 28 March 2022) which helps its users suffering from different orthopedic and neurological injuries to incorporate with their motor therapy for assistance at home. This proprietary tension system assists patients to perform finger extension following a grasp action. This fully functional, commercially available setup is a bewildering example of AI-based robot-assisted system for patients with motor disabilities.

Moreover, systems such as Microsoft Kinect play significant roles in physiotherapy and rehabilitation of stroke patients. Microsoft Kinect device (https://www.physio-pedia.com/The_emerging_role_of_Microsoft_Kinect_in_physiotherapy_rehabilitation_for_stroke_patients) (accessed on 28 March 2022) offers exciting and innovative ways of rehabilitation that make the treatment, and thus the subsequent adherence and motivation, more interactive and enjoyable. Microsoft Kinect allows stroke survivors suffering from motor disabilities to interact with an environment where they can perform different movement combinations without any need for a controller or attached device.

This review article provides an overview of, and deep insights into, modern alternative rehabilitation technologies. Moreover, the review article focuses on the importance of stroke rehabilitation while narratively explaining the socio-economic burden of this disease and related risk factors. Considering the increasing popularity and evidence of the benefits of technology-aided rehabilitation approaches, some commonly used stroke therapies to regain muscle activity are discussed. The reader can refer to the cited articles for more information.

Despite all presented discussions, we cannot deny the fact that the future is strongly associated with excess use of artificial intelligence in the field of rehabilitation—whether it is telerehabilitation, virtual reality, robot-assisted therapies, or participatory involvement of multiple techniques. Hopefully, I have answered the question. For more details, please refer to the cited article [2,3,4,5].

## Data Availability

Data presented in the paper is extracted from published work. The authors of the current study do not have the raw data.
